# A Pilot Study to Assess the Effect of Coaching on Emergency Nurses’ Stress Management

**DOI:** 10.3390/nursrep13010019

**Published:** 2023-02-01

**Authors:** Rabia Chahbounia, Abdellah Gantare

**Affiliations:** Laboratory of Health Sciences and Technologies, Higher Institute of Health Sciences, Hassan First University of Settat, Settat 26000, Morocco

**Keywords:** emergency nurses, transtheoretical coaching model, occupational stress, management

## Abstract

(1) Background: Emergency nurses are more exposed to a wider range of stressors, resulting in higher levels of burnout, reducing the quality of nursing care, and decreasing job satisfaction compared with other peers in other nursing departments. The objective of the current pilot research is to evaluate the efficiency of a transtheoretical coaching model on emergency nurses’ occupational stress management through a coaching intervention. (2) Materials and Methods: An interview, Karasek’s stress questionnaire, the Maslach Burnout Inventory (MBI), an observation grid, and a one-group Pre-test–Post-test questionnaire was carried out to evaluate the changes in emergency nurses’ knowledge and their ability to manage stress before and after attending the coaching intervention. A total of seven emergency room nurses at the proximity public hospital of the Settat area in Morocco have taken part in this study. (3) Results: The results have shown that all emergency nurses were subject to the job strain and iso-strain; four nurses were in moderate burnout, only one nurse was found in high burnout, and two were in low burnout. There was a significant difference between mean Pre- and Post-test scores (*p* = 0.016). Nurses’ mean score has improved by 2.86 points after attending the four sessions coaching experience, passing from 3.71 in the Pre-test to 6.57 in the Post-test. (4) Conclusions: The coaching intervention through a transtheoretical coaching model could potentially be an efficient strategy for enhancing the nurses’ knowledge and skills in stress management.

## 1. Introduction

At the international level, health care is a demanding field. According to the WHO, in both developed and under-developed countries, the occupational health issues of health-care workers due to stressful working conditions perform poorly in the quality of care, patient safety, medical errors, etc. In this perspective, it has been found in Great Britain and the United States that the annual cost of occupational illnesses and injuries in the health-care sector is the highest of all sectors [1]. In this context, Emergency Departments are a source of high stress for patients and clinicians.

As part of the clinical team and compared to their peers in other Nursing Departments, emergency room nurses are on a daily basis more exposed to a wider range of stressors and unpredictable work conditions. Therefore, they are subject to hypertension, stress cycle, depression, anxiety, high level of burnout, and so forth [2], which reduces the quality of nursing care, work engagement and abilities [2]. Research has identified several causes of stress within the Emergency Department, notably interpersonal conflicts between colleagues [3], constant orders from superiors, lack of human resources, highly demanding routines to deal with potentially traumatizing situations [3], suicide [3], a heavy workload, witnessing death and suffering of patients [3], and experiencing or witnessing violence against staff [3].

One study conducted by Chakhtoura Khalid in Morocco as a country in development, located in the north of Africa, has shown that nurses working in demanding services such as emergency room nurses had a higher degree of stress than those at the medical service, which correlates strongly with other results [4]. According to Abderrahim El Bouazzaoui, 32.4% of nurses working in emergency rooms had a higher level of emotional exhaustion [5].

Potentially, developing coping strategies to use in stressful situations requires nursing skills [3]. Among the predominant coping strategies used to manage stress are: planful problem solving (such as step-by-step planning and coming up with different solutions), positive reappraisal (self-improvements, such as finding what was positive in an experience and what was learnt, turning to spiritual approaches, and rediscovering value in the experience) [3], mindfulness meditation [6], and coaching, which offers a tool to enhance nurses’ competencies, both individually and collectively, and it helps the team to improve communication and avoid psychological concerns through managing their work-related stress and assisting them in developing their stress management skills. The emphasis is on individual needs, strengths, and shortcomings by dialogue and reflection in a space of confidentiality and trust [7]. Although there is a great diversity of coaching theories, practices, and strategies, it is still difficult to prejudge the effectiveness of one compared to another [8].

For this reason, in order to better manage the stress of emergency nurses, we have conducted a coaching intervention guided by a transtheoretical coaching model that integrates the most scientifically relevant existing coaching models which have a theoretical and ethical basis.

## 2. Materials and Methods

### 2.1. Study Design

**Figure 1:**
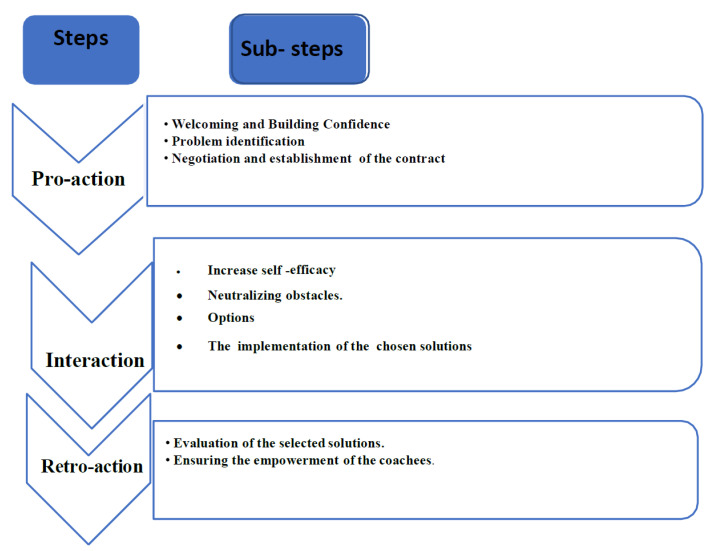
The steps of the transtheoretical coaching model.

A pilot study was conducted using a multiple-case replication study design. Seven emergency room nurses at a proximity hospital in the Settat area in Morocco were recruited for the study, which aimed to evaluate whether a coaching intervention, guided by three steps of a transtheoretical coaching model (Pro-action step, Interaction step and Retro-action step) (Figure 1), can be an efficient strategy to manage stress and avoid burnout among health-care providers working in an Emergency Department. Through the intervention process, the participants were asked to complete a series of instruments. 

An interview: The interview was divided into two sections: (1) socio-demographic characteristics: age, gender, marital status, number of years of work; (2) GROW model questions (Goal, Reality, Option and Will), which help to structure coaching sessions with participants.

Karasek’s stress questionnaire: The questionnaire was designed to measure social and psychological aspects of jobs.

Maslach Burnout Inventory (MBI): This inventory was designed to study the frequency of burnout (BO) and to evaluate the three elements of burnout (emotional exhaustion, depersonalization, and personal accomplishment).

An observation grid: The grid was designed to study the signs of the external behavior that translate the inner state experienced by the person and calibrate his/her behavior.

Pre- and Post-test: These tests are used to evaluate the nurses’ knowledge and skills in stress management before and after attending the coaching intervention.

### 2.2. Study Setting and Participants

This quasi-experimental study was carried out from 10 June 10 2022 to 17 July 2022; it was conducted with nurses working in the Emergency Department in a quiet hall at the proximity hospital in the Settat area.

The 7 nurses were invited to participate in the coaching sessions conducted according to the three steps (Pro-action, Interaction and Retro-action). After being informed that the participation was voluntary and that confidentiality of their information would be secured, all nurses volunteered to participate. Hence, the response rate obtained was 100%.

### 2.3. Transtheoretical Coaching Model

In an article (to be published), the transtheoretical coaching model, also known as an integrative coaching model, intended to be implemented in a stressful structure in order to accompany nurses under stress and avoid the other occupational health issues [9]. 

This transtheoretical coaching model (Figure 1) has three steps (Pro-action step, Interaction step, and Retro-action step). Each step is subdivided into sub-steps (Figure 1) inspired by Abdellah Gantare’s process [10]. The mentioned model has been synthesized from rigorous coaching models (Dojo model, PAIR model, ABC model, GROW model, PRACTICE model, and Peer Coaching Model) selected on an international scale following a critical analysis of coaching practices which consists of pointing out only the coaching models which are endowed with two scientific criteria. The first criteria relates to the theoretical bases while, the second one relates to the ethical bases. The coaching models that paves the way to the transtheoretical model are presented below:

The Dojo coaching model: Developed by Bernard Hévin and Jane Turner, it is composed of two models: (1) the disciplinary model, which refers to the work of general semantics [11] (p. 19), constructivism, the human relations movement [11] (p. 19) etc.; (2) the relational model, which refers to humanistic psychology and the human potential movement [11] (p. 20), etc.

The disciplinary model consists of evaluating the coachee’s unique situation using three specific skills of the coach:

- Observe: To take note of the coachee’s reality [12] (pp. 114–115).
- Accompany: To lead the client through a predetermined number of transformational steps, so that he can develop his life project [12] (p. 116).
- Empowering: To make the coachee aware that there are a set number of tasks that must be completed before creating and executing his life project [12] (p. 117).

The relational model involves four phases that walking alongside the coachee as they pursue autonomy:

- Understanding the request of the coachee [12] (p. 120).
- Listening for the establishment of a cooperative relationship with the client [12] (p. 120).
- Identify the current situation and the objective of the coachee [12] (p. 120).
- Clarify and stabilize a context in which the coachee can regain or even gain Autonomy [12] (p. 120).

The PAIR model: (Presence, Agreement, Intervention, and Review) belongs to the work of Sally Denham, Vaughan and Mark Gawlinski. It underlies the gestalt approach [13].

ABC Model: Ellis defined the ABC as follows: “A” represents the “Activating Event” (experience) that disturbs the individual. “B” represents the “Beliefs” (rational or realistic) about the activating event; and “C” represents the “Consequences” of the thought and beliefs [14] (p. 439). This model was adapted from Rational Emotive Behavior therapy introduced by Neenan and Dryden (2002) and developed by Albert Ellis in the 1950s and early 1960s [14] (p. 438).

GROW Model: Developed by Graham Alexander, it was later popularized by Sir John Whitmore [15] (p. 36). It refers to cognitive behavioral therapy (CBT). The steps of the GROW model are: Goal setting, Reality, Option, and Will [16] (p. 51).

PRACTICE model: The PRACTICE model was developed by Stephen Palmer (2007) as an adaptation of Wasik’s (1984) approach [17] (p. 131). The steps of this model are in the order of seven steps: (1) “Problem identification” [17,18] (p. 131), (2) “Realistic goals” [17,18] (p. 131), (3) “Alternative solutions generated” [17,18] (p. 131), (4) “Consideration of consequences” [17,18] (p. 131) , (5) ”Target solutions” [17,18] (p. 131)., (6) “Implementation of chosen solutions” [17,18] (p. 131), and (7) “Evaluation of the adopted action” [17,18] (p. 131).

Peer coaching model: From Varey (2002) [19] (p. 175), this model refers to solution-focused therapy, the person-centered approach, the cognitive behavioral approach, and learning theory [20] (p. 293). The steps of this model follow: Assessment and trust building, Planning, Formalizing process and scope, Defining purpose and goals, Clarifying facts and assumptions, Exploring possibilities, Gaining commitment to actions, and Offering support and accountability [19] (p. 173).

### 2.4. Measures

The instruments were used according to 3 steps of our transtheoretical coaching model (Pro-action step, Interaction step and Retro-action step).

#### 2.4.1. An Interview

The interview was divided into two sections: (1) socio-demographic characteristics, including age, gender, marital status, and number of years of work, and the (2) GROW model questions, which were used according to the 3 steps (Pro-action, Interaction and Retro-action).

In order to ensure that all the participants received a similar coaching session, the same questions were asked in all coaching steps, and one coach carried out all coaching sessions. There were four groups of questions that the coach asked, the details of each question can be found in Table 1 as follows:

#### 2.4.2. The Karasek Job Content Questionnaire

The Karasek Job Content Questionnaire was used at the pro-action step in its French version. It is an instrument designed to measure social and psychological aspects of jobs. It consists of 26 questions composing three subscales: evaluate psychological demands (9 items), decision latitude (9 items), and social support (8 items). The responses were given on a Likert-type scale: “Not at all agree “, “strongly disagree”, “disagree”, “agree”, and “strongly agree” [21]. The person is considered to be in “job strain” or “tension at work” when there is a combination of low latitude/high demand [22]. If the psychological demand score is higher than 20 and the decision latitude score is lower than 71, then the person is in the “tense quadrant” [22].

“Iso-strain” characterizes a situation that combines “job strain” and “isolation”: that is to say, an accumulation of a score higher than 20 of psychological demand with a score of decision latitude lower than 71 and a score of social support lower than 24 [22].

#### 2.4.3. The Maslach Burnout Inventory (MBI)

The Maslach Burnout Inventory (MBI) was used at the pro-action step. The Maslach Burnout Inventory (MBI) in its French version was used in order to study the frequency of burnout (BO) and to evaluate the three elements of the burn-out syndrome in our sample, including emotional exhaustion, depersonalization, and personal accomplishment. The original version of this scale is composed of 22 items that explore 3 dimensions: emotional exhaustion (EE) (9 items), depersonalization (DP) (5 items) and personal accomplishment (8 items) [23] (p. 193). Responses are given on a 7-point frequency scale, ranging from “never” to “every day” [23] (p. 193).

The score obtained from each dimension allows us to evaluate the degree of severity (low, moderate, high). Emotional exhaustion is considered low if the score is below 17, moderate if the score is between 18 and 29, and high if the score is above 30 [24]. The depersonalization is considered low if the total is less than 5, moderate for a total between 6 and 11, and high for a total higher than 12 [24].

For personal accomplishment, is considered low if the score is higher than 40, moderate if the score is between 34 and 39 [24], and high if the score is lower than 33 [24]. A high score for emotional exhaustion or depersonalization, or a low score for personal accomplishment, is sufficient to define BO [24].

The BO is said to be “low” if one of the three dimensions is pathological [24], “moderate” if two of the three dimensions are pathological and “high” if all three dimensions are pathological [24].

For the purposes of this study, we considered people with at least one pathological dimension to be professionally exhausted. BO is considered high (or severe) if emotional exhaustion and depersonalization are high with low personal accomplishment.

#### 2.4.4. The Observation Grid

The observation grid (Table A1) was used at the interaction stage, it inspired by the book *NLP* of Catherine Cudicio in order to better calibrate the behavior through the sensory mode VAKOG (Visual, Auditory, Kinesthetic and sometimes Olfactory or Gustatory representations) of the participants; by studying the signs of the external behavior that translate the inner state experienced by the person, by identifying the sensory register of the person from his non-verbal behavior and choice of words, gesture, posture, facial expression [25] (p. 28), and the quality of the voice (tone, rhythm of the speech, etc.) [25] (p. 28).

#### 2.4.5. Pre-Test–Post-Test

A Pre-test was conducted to assess nurses’ knowledge and skills on the effects and management of stress before attending the coaching intervention at the pro-action step (Table 2). The questionnaire is composed of seven items:

- Three items (2, 3, 4) concerning the nurses’ knowledge and the awareness of the stress’s effects.
- Four items (1, 5, 6, and 7) concerning the nurses’ stress management skills.

For each statement, participants were asked to answer “yes” or “no”. The Pre-test took approximately 10 min to complete.

The Pre–Post-test used in the current study was designed specifically by the authors and was inspired by the extant literature about the knowledge of stress’s effects and management skills.

The three items (2,3, 4) about the nurses’ knowledge and the awareness of the stress’s effects serve to explore their knowledge, as the way they interpret what happens to them is more determinant than the facts themselves in order to create the awareness and the need for attention to the risks associated with harmful stress [26] (p. 16), increase motivation and increase self-efficacy to cope with stressful concerns [26] (p. 16). The three items were developed based on Dale Carnegie’s book entitled *Comment dominer le stress et les soucis/How to deal with stress and worries* [26] (p. 16).

The four items (1, 5, 6, 7) concerning the nurses’ stress management skills explore the acquisitions of necessary skills for each person’s practice to deal with stress. The development of the four items was inspired by Aaron T. Beck’s automatic thought [27] (p. 50), Ivan Pavlov’s conditioning experiments [27] (pp. 78–79), and Willis Carrier’s work [26] (p. 41).

Aaron T. Beck was the founder of cognitive behavioral therapy, who highlighted the existence of automatic thoughts and outlined a way to deal with negative thoughts through “Defusing negative thoughts” [28] (pp. 7–8). Ivan Pavlov’s conditioning experiments are at the center of behavioral theories; he showed that the stimulus generates a reflex reaction, he impacted the technique “Resource anchoring” (associating a state of calm with a state of stress) in Neuro-Linguistic Programming [27] (pp. 78–79).

Willis Carrier’s work focused on personal development adapted to the business world. Willis Carrier was inspired by the founder of psychology William James, especially in the application of psychological principles to solve problems of human experience. In this regard, the emphasis is on how to put a stressful situation into perspective [26] (p. 41).

After the retroaction step, a Post-test was conducted to evaluate whether there were any changes in the nurses’ knowledge and skills on the effects and management of stress after attending the coaching intervention. The Post-test used the same questions included in the Pre-test. It also took about 10 min to complete. For each completed questionnaire, the Pre-and Post-test total scores were tallied. A score was given to each answer; answers with “yes” were given the score of one, whereas, answers with “no” were given the score 0.

### 2.5. Procedures

To carry out our study, an individual coaching intervention with each participant included 4 coaching sessions conducted according to three steps of the transtheoretical coaching model (Pro-action step, Interaction step and Retro-action step).

The Pro-action step includes one session, the Interaction step includes two sessions and the Retro-action step includes one session. 

The coaching sessions were held between 10 June 2021 and 17 July 2022 in a quiet hall at the proximity hospital. Each session lasted between 45 min and 1 h 15 min, and the sessions were spaced out over one week.

One week before the scheduled coaching intervention, every day, we made a visit to the Emergency Department to meet the nurses in order to talk to them about the objective of our study and have their agreement to participate in the study, the environment (a quiet hall) and the equipment required for the coaching experience were prepared in consultation with the hospital’s director and the nurses.

#### 2.5.1. Pro-Action Step: Scenario of Session 1

The Pro-action step includes three sub-steps: Welcoming and confidence building–Problem Identification–Negotiation and making contract.

- Welcoming and confidence building: For each participant, we started session 1 of coaching with “welcoming and confidence building” for the participant; then, he/she was asked about his/her experience of working in the Emergency Department.
- Problem identification: After “welcoming and building confidence” for our participants, we asked each participant guided by our interview guide (see Table 1 above) about his/her: age, gender, marital status, and number of years worked; then, each participant was asked about his/her goals in terms of managing his /her work-related stress: By asking him/her the following questions:

In your work, what makes you stressed?

What do you want to achieve in this session? How would you like to feel later?

Then, the participants were requested to complete Karasek’s stress questionnaire (which lasted for about 10/15 min), Maslach Burnout Inventory (which lasted for about 10 min) and the Pre -test-questionnaire.

Participants were informed that they will obtain feedback about the results of the questionnaires that they were asked to complete.

➢ Socio-demographic data:

The nurses’ mean age was 37.42 years, ranging from 22 to 61 years. Six of them were females and only one was a male, 5 nurses had between 6 and 15 years of experience, and 1 nurse had only one year of experience.

➢ Summary about job experience and goals of the participants

Participant 1:

Participant 1 said: «During my experience of emergency work, I had an increase in blood pressure (hypertension), I feel exhausted at the end of my work day…». «Currently, I find difficulty in the execution of some tasks delegated to me, in fact I feared that something bad happens to my patients during the execution of my work … I would like to work calmly without thinking too much in this way».

Participant 2:

Participant 2 said, «I have given enough to my work, I feel monotony, I feel too much stressed and as a result I have a heart rhythm disorder (tachycardia)…I always have the idea of doing a good job, … I can’t imagine that one day they will criticize my work, and because of these I find myself in conflict with my colleagues …I would like to focus on my work, without reflecting on the point of view of others».

Participant 3:

Participant 3 said, «I have problems with my work schedule, I feel stressed all the time, I can’t balance my personal and professional life…, I have two children, when I come to work, I leave my children at home..., I always think that something bad will happen to my children.»

Participant 4:

Participant 4 said about the work: «I work calmly, except that I find the work schedule restrictive…I try to control my stress; it is rare that I get stressed because of the lack of material... I would like to be coached on stress management, it will help me in my personal and my professional life.»

Participant 5:

Participant 5 said, «I feel insecure and stressed, especially when I work at night…, I can’t welcome patients alone, I always need a security guard. I am afraid to meet criminals or delinquents…I wish I could get rid of this stress and control this situation».

Participant 6:

Participant 6 said: «During my work, I hardly do the tasks ..., in fact, I am afraid that something bad happens to my patients during the delivery of the care requested by the physician.»

Participant 7:

Participant 7 said «I have an obsession diagnosed by a psychiatrist, I can’t give injections to my patients, I always need my coworkers to do it, I feel frustrated and too stressed when they talk behind my back, they say I’m incompetent…I am null I would like to change the work department, I would like to better control my thoughts about what they say.»

➢ Feedback about the nurses identified problems

Feedback was given to the participants about their identified problems and the results of the questionnaires (Karasek Job Content Questionnaire and Maslach Burnout Inventory).

All participants were informed that they were found to have job strain and iso-strain.

Participant 3 was informed that he was found in high burnout, participants 1, 2, 5, and 7 were informed that they were found in moderate burnout, and participants 4, and 6 were informed that they were found in low burnout (see Table 3).

- *Negotiation and making contract:* After knowing the objectives of our participants, we made a contract with them about the place of coaching (a quiet hall in the hospital), the topic of coaching (stress management), the number and frequency of sessions (4 sessions, spaced in a week), and the tools that will be used (coaching exercises in stress management, observation grid, etc.)

#### 2.5.2. Interaction Step: (Scenario of Session 2 and Session 3)

The interaction was carried out in two sessions (session 2 and session 3).

It included three sub-steps: Increase self- efficacy, Neutralizing obstacles and options, and Implementation of the chosen solutions.

After the negotiation and the making of the contract, and before undertaking the coaching exercises in stress management, as a result of the high level of burnout found in participant 3, it was suggested that she consult another experienced and specialized person, such as a psychologist or a psychiatrist, to treat the burnout.

- Increase self-efficacy–Neutralizing obstacles and options:

Each participant received coaching exercises based on Neuro-Linguistic Programming (Table 4). Each exercise lasted about 15 min. At the same time, we tried to increase their self-efficacy, to observe, to calibrate the behaviors, and to know the VAKOG system of all the participants using the observation grid (Table A1) in order to better interact with them using different ways (for example: We have used different videos and presentations to attract the attention of the participants with the visual VAKOG system.

In this perspective, at the beginning of the coaching process, participant 4 was inattentive and very pressed for time; after the calibration of his behaviors and synchronizing with him, he became cooperative. It was also observed that participant 3 was very tired. For this reason, it was decided to postpone the coaching session for another time.

Participant 2 was very sad and anxious; we tried to be active listeners and more empathetic with her.

- Implementation of the chosen solutions:

At the end of this session, each participant was asked the following questions guided by our interview guide of the GROW coaching model (Table 1 above): “What are the possible options? What solutions have you chosen in similar situations? Could the intervention help you going forward? How did you find your experience of attending coaching sessions?”

#### 2.5.3. Retro-Action Step (Session 4)

The Retro-action step includes two sub-steps: Evaluation of the selected solutions and Ensuring the empowerment of the coachees.

- Evaluation of the selected solutions: At this session, a Post-test was conducted to evaluate whether there were any changes in the nurses’ ability to manage stress.
- Ensuring the empowerment of the coaches: In order to ensure the autonomy and the empowerment of nurses in their ability to manage stress, each participant was asked the following questions (guided by the interview guide of GROW coaching model in Table 1): “How did you find your experience of attending coaching sessions?” In addition, we asked them if there was another subject they would like to be coached on by asking each participant this question: “Do you have another issue that bothers you, and that you would like to have coached on?”

### 2.6. Data Analysis

Statistical analysis related to the Pre–Post-test, Maslach Burnout Inventory, and Karasek’s stress questionnaire was performed with the statistical software SPSS version 20 software.

The paired-sample Wilcoxon test was used to compare mean Pre-and Post-test scores. The significance level was set to 0.05 (α = 5%).

Descriptive statistics were used to calculate the range (maximum and minimum), mean, median, and standard deviation of Pre-test and Post-test scores.

### 2.7. Ethical Considerations

This study adheres to the principles outlined in the Declaration of Helsinki. Verbal informed consent was obtained from all the nurses to participate in the study and for using the data. Ethical approval was obtained from the Institutional Review Board (IRB) of the Moroccan Association for Research and Ethics, Research Ethics Committee (IRB00012973 Moroccan Association for Research and Ethics IRB #1). [No.11/REC/21] on 27 September 2021.

## 3. Results

### 3.1. Stress and Burnout among Participants

The results of Karasek’s stress questionnaire revealed that all emergency nurses were found to have job strain and iso-strain.

By analyzing the results of Maslach Burnout Inventory, only one nurse was found in high burnout, while four nurses were in moderate burnout, and two were in low burnout (Table 3).

### 3.2. Pre–Post-Test Results

The results of the Pre-test and the Post-test showed that the highest score accounted was 6 in the Pre-test and 7 in the Post-test, while the lowest score was 2 in the Pre-test and 6 in the Post-test (Table 5). The nurses’ mean score has improved by 2.86 points after attending the coaching experience, passing from 3.71 in the Pre-test to 6.57 in the Post-test.

## 4. Discussion

It is of crucial importance to prevent and limit the negative consequences of emergency nurses’ occupational stress [29] in order to avoid the increased irritability, emotional exhaustion, and decreased job satisfaction, which affects the quality of nursing care, work engagement and leads to reduced abilities [30].

For this reason, the coaching of nurses is necessary to prevent and limit these negative consequences of stress despite the variety in the theories and the practices of coaching, which causes difficulty in knowing which practice is more effective than others [8]. 

The present current study was completed to describe and to examine whether the coaching of nurses guided by a transtheoretical coaching model (which was based on different coaching models and theories such as constructivism, humanistic psychology, gestalt approach, cognitive behavioral therapy, solution-focused therapy, learning theory, etc.), can be an effective strategy to enhance the nurses’ knowledge on the effects of stress and the management skills stress.

As anticipated, positive changes in the post-questionnaire test have been noted after receiving the coaching intervention guided by the aforementioned model. This reflects that the coaching through the transtheoretical coaching model was a valuable strategy to enhance the nurses’ knowledge on the effects of stress and the management skills stress.

In Morocco, existing literature on the topic did not reveal any previous research that has suggested the possibility of using a transtheoretical coaching model that is derived from different theoretical and conceptual foundations of coaching as a strategy dedicated specifically in order to improve nurses’ knowledge and management skills about stress.

In this regard, Chesak et al. showed the common types of stress management interventions dedicated specifically to nurses: psychological skills training (based on coping/stress/resilience training; cognitive behavioral therapy; mindfulness-based, and meditation training), care model, massage, energy therapy (healing touch, jin shin jyutsu, reiki), acupuncture/acupressure aromatherapy, yoga, communication training, multimodal interventions, and other intervention types (e.g., professional identity program, quiet time, hardiness education).

On the one hand, Alkhawaldeh et al. showed in their studies that only the cognitive behavioral skills training with mindfulness-based intervention was more effective in reducing occupational stress among intensive and critical care unit nurses [31].

On the other hand, the study carried out by Maslakpak et al. in 2016 has also shown that the use only of Neuro-Linguistic Programming can increase coping with stressful situations, and it can reduce the adverse effects of occupational stress [32].

### Limitation

The present study has certain limitations that should be addressed: in particular, the restricted number of subjects involved in the study. This can be explicated by the fact that the coaching intervention was incorporated into a single-center study within an Emergency Department that has only seven of the staff.

Therefore, the findings have limited transferability because emergency nurses from the proximity hospital in the Settat area of Morocco were conveniently sampled. Hence, there is a need for further multicenter studies about this topic to confirm our findings.

Thus, the Pre–Post questionnaire and the observation grid used in this study were self-developed by the authors based on the literature review, and its content was not subject to validation by experts. Hence, it appears to be necessary to address it to experts for validation to ensure its reliability.

Furthermore, various constraints were faced in terms of the lack of psychiatrists and psychologists and long-term follow-up of the participants.

## 5. Conclusions

This study demonstrates the efficacy of a coaching intervention based on a transtheoretical coaching model on nurse’s knowledge of the effects of stress and stress management skills. 

This finding is of paramount importance because this study is the first of its kind to deal with such a subject. In addition, a few studies have suggested a few strategies to cope with the stress of emergency nurses. Yet, the proposed strategy was conducted according to a few participants’ own stressful situations. Hence, there is a need to conduct further multicenter studies to assess and transfer this transtheoretical coaching model that seeks to handle the various stress situations.

## Figures and Tables

**Table 1:** Questions of GROW model used according to the coaching steps.

Step of Coaching	Questions (GROW Model)
Pro-action step	Goal: What do you want to achieve in this session [16] (p. 50)? Afterward, how would you like to feel [16] (p. 50)?
Interaction step	Reality: How did you find your experience of attending coaching sessions [16] (p. 53)? How were you involved in it? What didn’t work [16] (p. 53)? Options: What are the potential solutions? What solutions have you chosen in similar situations [16] (p. 53)?
Retro-action step	Will: Who could possibly be able to help you [16] (p. 53)? How devoted are you to carrying out the actions we assign [16] (p. 53)?

**Table 2:** Pre–Post-Test Questionnaire.

Item	Yes	No
1. I am able to control my stress		
2. I pay attention to my body’s physical reactions to cope with stress		
3. I know the difference between positive and negative stress		
4. I know that stress affects the body		
5. I know how to get rid of the negative thoughts that come to my mind		
6. I know how to associate a state of calm with a state of external stress		
7. I know how to relativize a stressful situation		

**Table 3:** Burnout dimensions and intensity.

Participants	Three Dimensions of Burnout						Intensity of Burnout
	Score of Emotional Exhaustion	Intensity of Emotional Exhaustion	Score of Depersonalization	Intensity of Depersonalization	Score of Personal Accomplishment	Intensity of Personal Accomplishment	
Participant 1	38	High	12	High	34	Moderate	Moderate
Participant 2	46	High	14	High	36	Moderate	Moderate
Participant 3	45	High	19	High	42	Low	High
Participant 4	14	Low	00	Low	39	Moderate	Low
Participant 5	41	High	11	Moderate	41	Low	Moderate
Participant 6	30	High	00	Low	29	High	Low
Participant 7	48	High	23	High	39	Moderate	Moderate

**Table 4:** An overview of the coaching exercises for stress management.

Exercise 1: Resource Anchoring (Resource anchoring is a Neuro-Linguistic Programming technique which refers to the process of associating an internal response with some external or internal trigger so that the response may be quickly, and sometime covertly, re-accessed. This technique is based on Pavlov’s conditioning experiments where a stimulus generates a reflex reaction [27] (pp. 78–79). The use of this technique was tailored to the coach’s goals (to associate a state of calm with a state of stress).) (Participants’ goal: Associate a state of calm with a state of external stress)	How does your body react to stress? When you get stressed, it is your shoulder that tenses up; so when you get stressed, pay attention to your shoulders, overplay this tense up (tense up even more), then take a deep breath and relax, repeat the same exercise each time you are stressed until it becomes a reflex so that the body learns to automatically self-calm.
Exercise 2: Defusing participants’ negative automatic thoughts (Defusing negative automatic thoughts were established from the book entitled «*La Boîte à outils de la gestion du stress/ the stress management toolbox*» [27] (p. 50), inspired by Aaron T. Beck, the founder of Cognitive Behavioral Therapy who has highlighted the existence of “automatic thoughts” [28] (pp. 7–8) (the little voices inside some participants: «I feared that something bad happens to my patients during the execution of my work...». «Thinking that something bad will happen to my children...». «Thinking that I will meet criminals or delinquents in my workplace...». «Thinking I am null…».	To turn off that voice: Identify the thoughts that triggered the negative emotion - Sort out objective reality from distorted reality Identify the distortions Find the antidote, the thought liberator (e.g., Focus your attention on something like color to cut off this inner voice...)
Exercise 3: Putting a stressful situation into perspective (Putting a stressful situation into perspective was created by Willis Carrier, who has focused his research in personal development adapted to the business world; he was inspired by the founder of psychology William James. In this regard, William James taught his students how to resign themselves or solve problems of human experience, in order to accept the situation as it is), Willis was also inspired by Lin Yutang who found that “true serenity can only result from the acceptance of the inevitable” [26] (p. 41).	If I were an outside observer of the situation, what would I think? Assume the worst Prepare to accept the worst Learn from the worst

**Table 5:** Minimum, maximum, mean and standard deviation (SD) of Pre-test and Post-test scores (*n* = 7).

	Minimum	Maximum	Mean	SD
Pre-test	2.00	6.00	3.7143	1.25357
Post-test	6.00	7.00	6.5714	0.53452

A significant difference was observed between the Pre- and Post-test scores (Test Wilcoxon, *p* = 0.016). These results suggest that the nurses’ knowledge on the effects of stress and the management skills stress have changed after participating in the coaching experience.

## Data Availability

All relevant datasets in this study are described in the manuscript.

## Appendix A

**Table A1:** The observation grid of the behaviors observed in the participants during the coaching intervention.

Element Observed	Participant 1	Participant 2	Participant 3	Participant 4	Participant 5	Participant 6	Participant 7
How is his/her posture?	Comfortable posture; relaxed	She was very attentive	She looked tired	At the beginning of the coaching session, he was inattentive, he crossed his arms, very pressed for time	She nodded her head	Stiff posture but attentive	Relaxed posture attentive, friendly, cooperative
How it is the breathing rhythm	Medium and balanced breathing	Was fast, irregular	Slow breathing	Deep	Normal	Chest breathing, rapid	Slow abdominal breathing
How it is the tone of the voice	Steady voice with conversational distance	She spoke quickly with a high-pitched, agitated voice	Low	Cutting	She was calm	Fast voice long conversational distance	Vibrant and low voice Short conversational distance
What are the facial expressions	Serious, attentive	Expressive, sad, she is not satisfied with her current life	Caring	At the beginning of the process, he was embarrassed, then he became comfortable	Serious, caring, very glad to be coached	Inexpressive	Serious and curious
How are the eye movements	She looked to the sides and in front of her while searching for a sound	She looked at the top right (she thought of the past) Friendly, serious	She looked at the coach’s eyes (she was listening)	At the beginning, the look of the participant was averted; during the coaching process, he became cooperative by looking at the eyes of the coach.	She followed the coach with her eyes; she was very concentrated.	She looked at the coach’s eyes, she was attentive; also from time to time, her eyes are turned up, to	Eyes down to the left (which reflect the emotions, sensations), also she looks from
						the right and to the left.	time to time in front of her, looking for a sound (she was attentive)
Vocabulary used	This participant often repeated the following sentence: *«*I’m listening to you………. It’s very interesting*»*. She used a vocabulary that is part of the auditory VAKOG system.	She said: «I remember the past, it choked me... I feel that I have become very nervous, I would do a different job.*»*. She used a vocabulary that belongs to the kinesthetic VAKOG system.	She was very enthusiastic and cooperative she always asked toward the end of each session, what we are going to do at the next session. She often used: «I am listening to you... okay*»*. She used vocabulary of the auditory VAKOG system.	At the beginning of the first session, he wanted us to go very fast, he said «We need a lot of time, we must to go fast*»*; after synchronizing with him, he became cooperative and satisfied, he said: «It’s very interesting, I’m listening to you, we can take our time, the other things can be postponed». He frequently used the word *«*I see*»* He used a vocabulary of the auditory VAKOG system.	The vocabulary used corresponds to the visual and kinesthetic system, i.e., the participant often used *«*I see, I feel that it is useful...*».*	She said, «I see...It appears to me.*»* She has a visual VAKOG System.	She comments on the coaching process *«*it’s all good...It is beneficial.*».* She has a kinesthetic system
